# Open Debates Conducive for Vaccination Rate Flatlines: A Scoping Review and Convergent Cross Mapping

**DOI:** 10.3389/fpubh.2022.830933

**Published:** 2022-04-14

**Authors:** Zhiwen Hu, Ya Chen

**Affiliations:** School of Computer and Information Engineering, Zhejiang Gongshang University, Hangzhou, China

**Keywords:** COVID-19 pandemic, COVID-19 infodemic, digital health, vaccine passports, vaccine incentives, vaccine confidence, behavioral interventions, science-based policy

## Abstract

COVID-19 pandemic is fueling digital health transformation—accelerating innovations of digital health services, surveillance, and interventions, whereas hastening social contagion of deliberate infodemic. The USA and many other countries are experiencing a resurgent wave of the COVID-19 pandemic with vaccination rate slowdown, making policymaking fraught with challenges. Political leaders and scientists have publicly warned of a “pandemic of the unvaccinated,” reinforcing their calls for citizens to get jabs. However, some scientists accused elites of stigmatizing the unvaccinated people and undermining the moral pillars of public health. Following the PRISMA-ScR guidelines, we first reviewed the nuances of stakeholders involved in the ongoing debates and revealed the potential consequences of divisive pronouncements to provide perspectives to reframe extensible discussions. Then, we employed the convergent cross mapping (CCM) model to reveal the uncharted knock-on effects of the contentious tsunami in a stakeholders-oriented policymaking framework, coupled with rich metadata from the GDELT project and Google Trends. Our experimental findings suggest that current news coverage may shape the mindsets of the vaccines against the unvaccinated, thereby exacerbating the risk of dualistic antagonism in algorithmically infused societies. Finally, we briefly summarized how open debates are conducive to increasing vaccination rates and bolstering the outcomes of impending policies for pandemic preparedness.

## Introduction

In the wake of the COVID-19 pandemic, the World Health Organization (WHO) characterized COVID-19 infodemic as an overabundance of information—“some accurate and some not—that makes it hard for people to find trustworthy sources and reliable guidance when they need it” on 2 February 2020 (1). This assessment sheds light on the fact that we are struggling with both a pandemic and a co-evolving infodemic. Unfortunately, the dubbed “pandemic of the unvaccinated” has taken the epicenter of COVID-19 infodemic since July 2021.

Recently, the catchphrase “pandemic of the unvaccinated” has sparked a global debate. The USA experienced a surge in COVID-19 infection rates, raising the issue of community transmission, particularly among unvaccinated Americans in July. Federal officials, governors, and media elites successively complained that not enough Americans have rolled up their sleeves to get vaccinated. On 16 July 2021, Dr. Rochelle P. Walensky, director of the Centers for Disease Control and Prevention (CDC), phrased current COVID-19 as a “pandemic of the unvaccinated” at a press briefing (2). Meantime, she testified that over 97% of people entering U.S. hospitals with COVID-19 are unvaccinated. On the same day, U.S. President Joe Biden echoed Walensky's assessment at the White House, saying, “Look, the only pandemic we have is among the unvaccinated” (3). One week later, the American Society for Reproductive Medicine (ASRM) followed suit and recommended that vulnerable pregnant individuals in the USA avail themselves of vaccines, citing the preponderances of evidence (4, 5).

In the aftermath of that, many U.S. State Officials and State Medical Officers have repeatedly praised the plausible statement. On 23 July, Kay Ivey, Governor of Alabama, made her position clear: “It's time to start blaming the unvaccinated folks, not the regular folks. It's the unvaccinated folks that are letting us down.” According to recent statistics, Dr. Thomas Dobbs, State Medical Officer of Mississippi, stated on 9 August that 97% of positive cases were from unvaccinated population and 89% of those hospitalized are unvaccinated, along with 82% of deaths (6). Echoing Dr. Dobbs' message, Tate Reeves, the governor of Mississippi, acknowledged that the 4th wave of pandemic is becoming a “pandemic of the unvaccinated,” spurred by the surge of Delta variant in Mississippi (7). Despite the backlash of Delta variant, he emphasized that recent CDC guidance requiring Americans to wear masks regardless of vaccination status is foolish and harmful (8), and hence he would not enforce a statewide mask mandate.

According to our survey, such plausible statements are shaping global discourse, capturing the attention of English-language media outlets in more than 80 countries as of 30 November 2021 (9). For examples, Dr. Nicola Spurrier, Chief Public Health Officer for South Australia, pleaded with South Australians to get the jab to stop a “pandemic of the unvaccinated.” “You can't rely on the other 80 per cent having had their vaccine,” she urged on 3 September. As Europe's most populous country, Germany has been grappling with the 4th wave of COVID pandemic, along with a resurgence of hospital admission rate, vaccination rate flatlines, and high 7-day incidence rate since May 2021. Two months later, Jens Spahn, Health Minister of Germany, broke the silence and reiterated that his country was also experiencing a massive “pandemic of the unvaccinated” on a press (10).

Arguably, the blame game is proliferated by the ongoing tsunami of unreliable information. However, the potential consequences of such tsunami remain unknown. In the scoping review section, we will scrutinize the dissensions on the unvaccinated among scientists and politicians, and provide hallmark references to reframe extensible discussions. In the section of convergent cross mapping (CCM) experiment, we will unveil the potential effects on the audiences who consumed the related news coverage. In the conclusion, we will briefly summarize how all stakeholders can contribute their share to achieving societal consensus through open debates.

## Methods

### Scoping Review Protocols

**Figure 1:**
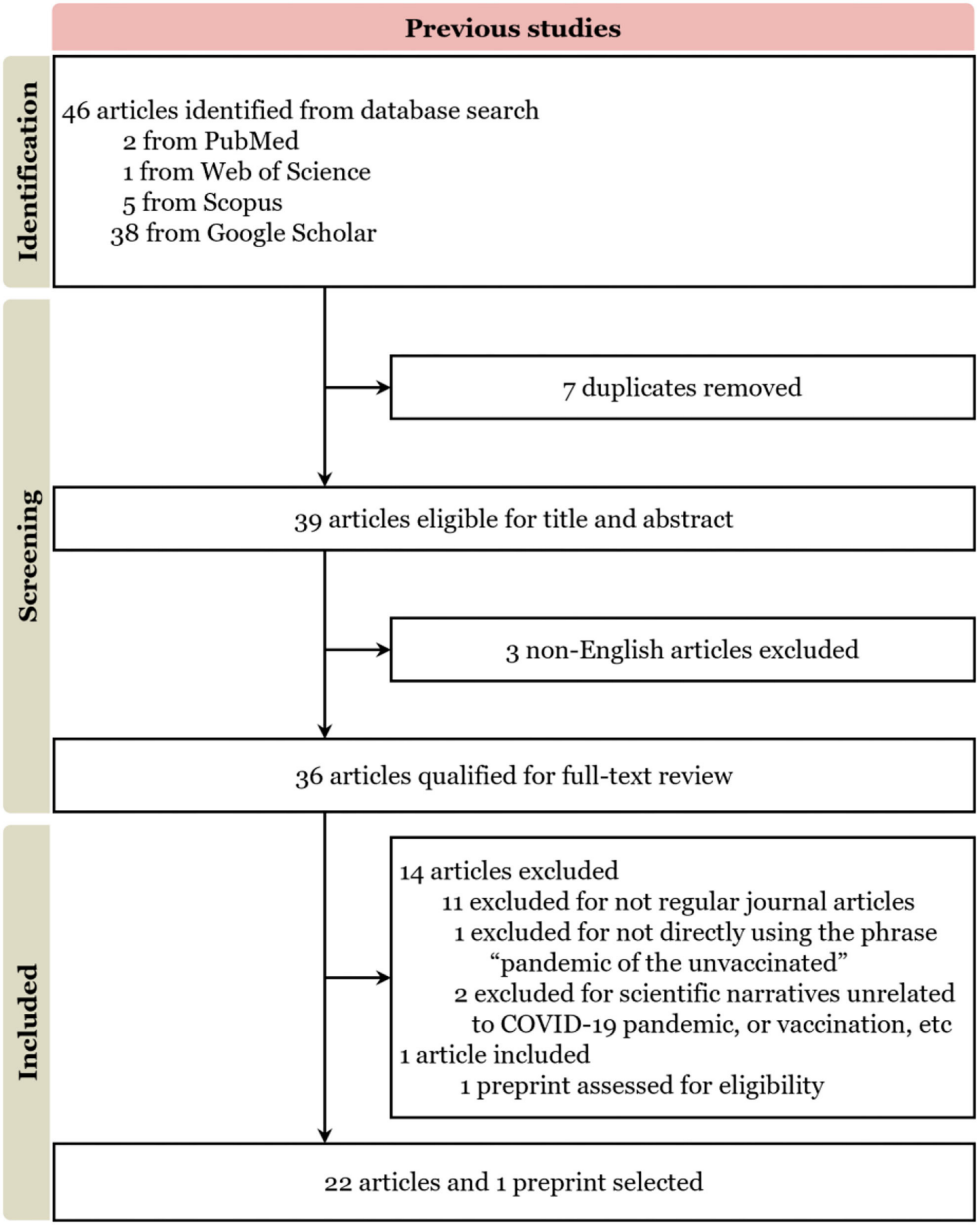
Literature selection process based on PRISMA-ScR. Supplementary Material gives a more detailed and formal explanation, for more details, see Supplementary Table 2 (Final reference list).

Studies exploring the use of “pandemic of the unvaccinated” relating to COVID-19 were reviewed by use of the scoping review methods proposed by Tricco et al. (11). We followed the scoping review protocols of the Preferred Reporting Items for Systematic reviews and Meta-Analyses extension for scoping reviews (PRISMA-ScR) (Figure 1).

**Search strategy and selection criteria**

We searched PubMed, Web of Science, Scopus, and Google Scholar to identify relevant English-language articles published between 16 July and 30 November 2021. Search terms included “pandemic of the unvaccinated” and “the unvaccinated” in combination with “COVID-19” and “Covid”. Only English-language papers were reviewed.

Concretely, exploratory searches were regularly done on Google and COVID-19 Open Research Dataset Challenge (CORD-19), MEDLINE, and PsycINFO from 16 July to 1 December 2021. These searches were not restricted by study design, and both peer-reviewed and gray literature were included. Then, we defined the Review scope, developed the research questions, and determined eligibility criteria. After such activity, PubMed, Web of Science, Scopus, and Google Scholar were selected for this Review because they include peer-reviewed literature in the fields of public health, behavioral sciences, psychology, clinical sciences, and public policy. Variations of the key search terms can be found in the panel.

In the wake of the COVID-19 pandemic, the scientific community offers rapid publication pipelines to accelerate COVID-related articles publishing, along with a large volume of research that was available before being published as non-peer-reviewed preprints or articles in press (12). We considered those pending-published works in the screening process. Additionally, we leveraged the co-citation and bibliographic coupling of Semantic Scholar and Connected Papers to cross validate the final reference list for screening, based on originality and relevance to the broad scope of this Review.

Two primary reviewer and a secondary reviewer screened the full texts of the 46 candidates. To minimize bias as much as we can, any disagreement among the reviewers was resolved by meeting and discussing within the team to reach a consensus. Finally, out of 46 articles identified, 22 articles and 1 preprint in English language were eligible based on exclusion criteria for a full-text review.

### Convergent Cross Mapping (CCM) Model

**Figure 2:**
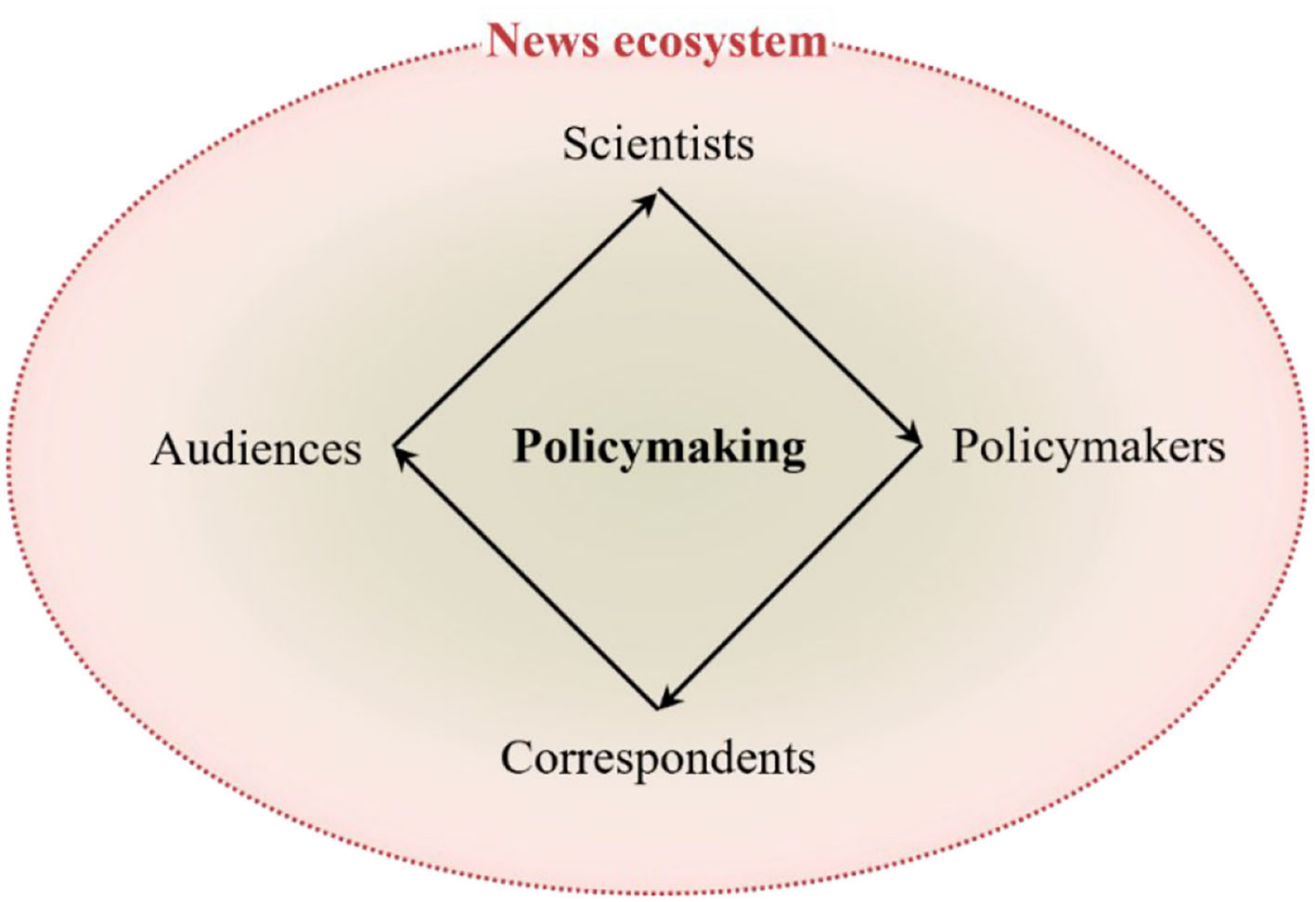
Under the umbrella of the news ecosystem, science-based policymaking is a cyclical process of scientific and societal consensus in which every scientist, policymaker, correspondent, or audience has a stake. Efficacious feedbacks between policy needs and gaps in scientific knowledge can underpin policy outcomes, with stakeholders contributing their share.

In a stakeholders-oriented policymaking framework, not only do scientists, policymakers, correspondents, and audiences influence one another in algorithmically infused societies, but their mindsets and beliefs are shaped by both the news ecosystem and policymaking processes (Figure 2) (13).

We characterized global news ecosystem as a complex system, and the evolution process of news ecosystem can be modeled as a dynamic process. The trajectory of evolution can be expressed as a manifold in a multidimensional space. News coverage system and news consuming system can be regarded as two subsystems of the whole news production-consuming ecosystem.

To demonstrate the dynamic relationships between the two subsystems, we employed the convergent cross mapping (CCM) model to detect mutual interactions. Concretely, the CCM model is a novel method for detecting mutual influence in coupled complex systems (14, 15). We orchestrate the query data on the phrase “pandemic of the unvaccinated” from GDELT project and Google Trends between 16 July and 30 November to demonstrate the interaction and causation paradigms between the two subsystems. The GDELT project retrieves 65 multilingual online news by leveraging the capacity of machine translation and neural network image recognition, sampling for 15 min per day (16). Meanwhile, Google Trends is an instant metric for collective search behaviors, since about 63% of users use Google to search for ubiquitous information (17). Therefore, the CCM model can examine the dynamic mutual interactions and coupled feedback effects both within and between the two subsystems with that rich metadata.

## Results

### Scientists Divide on Official Statements

In the scoping review, scientists have pledged to provide insightful commentary on the controversial topic—COVID-19 is becoming a “pandemic of the unvaccinated.” Neutrally, Frank Brodhead reported that top concerns of unvaccinated nursery staff were safety, including uncertainty about the vaccines' long-term effects, and mistrust of the vaccine development and approval process, according to a survey by the American Nurses Association (ANA) (18). Broadly, scientists divide on the immediate implications and long-term negative effects of such conclusion.

Some scientists endorsed the official rhetoric (19–23). Supporters concluded that the more virulent Delta variant posed a risk to the “pandemic of the unvaccinated” before the eventual attainment of herd immunity (24), particularly in-person schools (25), and Black, Indigenous, and People of Color (BIPOC) (26). Dr. Hooper strongly urged that healthcare workers (HCWs) should get their jabs in her editorial (27), as well as Kelly and Jackson (28). Dr. Akova, Editor-in-Chief of *Infectious Diseases and Clinical Microbiology*, applauded the statement in his editorial for its appropriateness, despite the fact that fully vaccinated populations could also become infected with high viral loads and there is not a one-size-fits-all evidence-based vaccination strategy (29). Concomitantly, Dr. Cohen, Editor of *The Milbank Quarterly*, commented that vaccine hesitancy in the US nudged COVID-19 into a pandemic of the unvaccinated, despite the availability of effective vaccines (30). Yu and colleagues strongly suggested that governments should persuade all citizens to take their shots since the present pandemic has evolved into an unvaccinated pandemic (31). Longhurst and Their concluded that interregional differences of vaccine uptake in USA were the primary cause of an unvaccinated pandemic (32). Radically, Dr. Franco, Editor-in-Chief of *BMJ Evidence-Based Medicine*, advocated for vaccine mandates to lift vaccination rate flatlines in high-income countries (HICs) with sufficient supply, as an essential component of preparedness against vaccine hesitancy and anti-vaxxer propaganda (33). Furthermore, Samaranayake and Fakhruddin concluded that the high efficacy of the approved COVID-19 vaccines, extrapolated from such statements made by some Western officials (34).

Instead, many scientists are far more skeptical. Outspoken opponents slammed such political contestation that excoriated the unvaccinated in absence of grounded evidence (35). As one of the first voices, Dr. Kampf urged that authorities of the USA and Germany put extra effort into society together rather than stigmatizing the unvaccinated (36). Insightfully, Dr. Moodley of Stellenbosch University, South Africa, argued that the false descriptions of COVID-19 as a “pandemic of the unvaccinated” or “a self-inflicted pandemic” are simply attributing the current outbreak to the unvaccinated, regardless of medical contraindications, and such stigma would further encourage selective treatment behaviors in low-and-middle-income countries (LMICs), against the medical doctrine of sacrificial expectations (37). Concomitantly, Olivier, Honorary Professor at University of the Free State in South Africa, denounced in a preprint that some authorities pitted the vaccinees against the unvaccinated with specious statements, regardless of the fact that the virus mutates in the vaccinees (38). Additionally, Dr. Garcia criticized that U.S. CDC concluded better understanding the biological-social implications due to systemic racism (39).

“Listen to the scientists”—some variation of this phrase is frequently used in debates over health care policy and practice. Such phrases emphasize the paramount importance of reaching scientific consensus, which necessitates the exchange of substantial information about vaccination. As a cautionary story, current debates may provide an opportunity to introspect how to empower scientists with more channels to convey trustworthy information (40), strengthen people's digital health literacy (or eHealth literacy) (41), eventually restore trust and bridge the divides (42).

### Better Together: Bridging the Bifurcation

According to the scoping review, reframing COVID-19 pandemic into a “pandemic of the unvaccinated” is scientifically inaccurate and vilifying to the unvaccinated. COVID-19 pandemic involves not merely the unvaccinated but all of us (43). Previous surveys have shown that the emerging SARS-CoV-2 variants are not fully sensitive to vaccines available, which beclouds any reasonable prediction. Today, extra bedside-to-bench efforts are required to better understand both antiviral efficacy and unwanted side effects of vaccines in the context of long COVID (44). Furthermore, new designated Variants of Concerns (VOCs) like Omicron (B.1.1.529) may further nudge vaccine hesitancy and exacerbate global vaccine imbalance (45).

Unfortunately, we are hitting compassion fatigue with the unvaccinated, irrespective of sociocultural and socioeconomic inequity (e.g., vaccine shortages in LMICs) (Only 2.5% of the people in LMICs are fully vaccinated, compared with 66% of the population in HICs.) (46). According to the Doherty Institute polls, 63% South Australians support businesses having the right to deny services to the unvaccinated, the highest rate in Australia. Dr. John Brayley, Chief psychiatrist of South Australia's Health, told the COVID-19 Response Committee (CRC) that the mental health fallout from the pandemic would be dealt with for 4–5 years (47).

Politicization and polarization of public health must remain vigilant (40). The looming worrying is partisan perceptions of political elites inadvertently feed the bias of impending policymaking (48–50). In many countries, a vaccination certificate policy—a certification reduces public health restrictions for their bearers—essentially functions as a mandatory vaccination program (51). But radicals take such claims and inform national mandatory vaccination policies before attainment of herd immunity (33, 52), lest legal and ethical perils lose their niches (53–55). Ethical judgments must be used to evaluate policy outcomes, take precedence over assumed exemptions (e.g., marginalized groups like refugees and migrants) (56). On 14 September 2021, Dr. Eric T. Payne wrote a 19-page letter to the College of Physicians & Surgeons of Alberta (CPSA) to complain that Walensky's declaration would perpetuate unneeded societal hatred and division (57). As a senior doctor, Payne may cost his job for fighting against the impending “medical tyranny” over mRNA vaccinations [the most controversial COVID-19 vaccines (58)]. One month later, Dr. Bonnie J. Fraser Henry, BC's Provincial Health Officer of Canada, issued a Public Health Order on COVID-19 vaccination that applies to health professionals (59). Notably, she exclusively depicted the “unvaccinated persons” as posing risk of harm to residents in this order.

Obviously, filling the niches of ethical perils could narrow the divide between scientists and politicians, and pave the way forward to inform science-based policy and flatten the curve of coronavirus.

### Knock-on Effects of the Dubbed “Pandemic of the Unvaccinated”

How we reap the benefits of tit-for-tat debates? And yet, little is known about the knock-on effects of shaping public perception and behaviors in current open debates. To enable richer representations beyond literature review, we introduce the CCM experiment to further uncover the knock-on effects in the feedback loops of the policy-making model, capture the reinforcing or dampening effects on social conformity, and demonstrate how important people's perceptions of “the unvaccinated” are.

**Figure 3:**
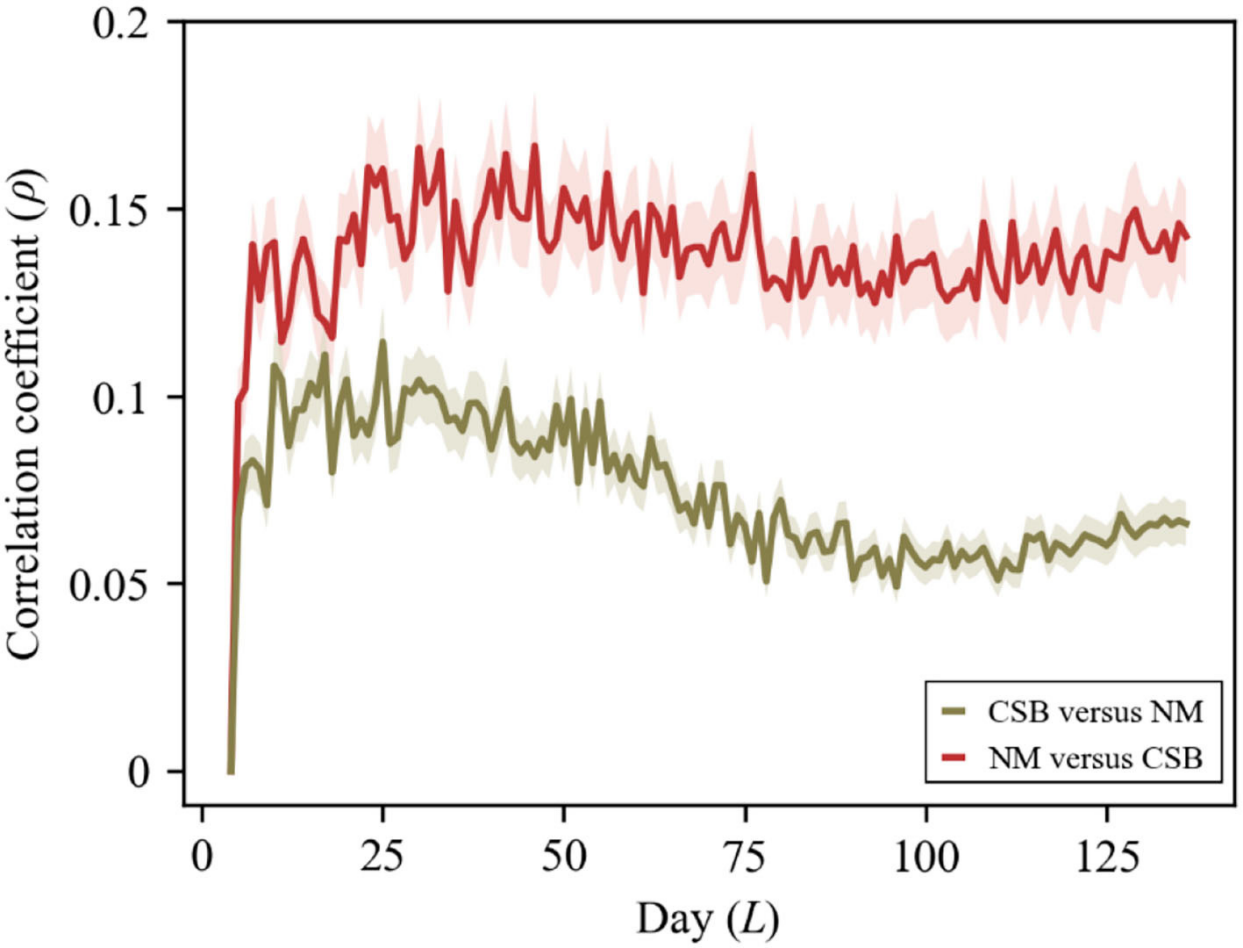
Detecting causation with convergent cross mapping (CCM) model. With convergence, the skill of cross-map estimates, indicated by the correlation coefficient (ρ), increases with the cumulative interactions of the following days between 16 July and 30 November 2021 (time series length *L* = 138). The patterns indicate that NM vs. CSB is more powerful than CSB vs. NM. For original experimental data, please see Supplementary Table 1 of the Supplementary Material.

Our experimental results suggest asymmetrical bidirectional coupling relationships between news media and collective search behaviors, which accords with previous findings (Figure 3) (14, 15). Moreover, news media (NM) has a stronger reinforcing influence on collective search behaviors (CSB) than the reverse, along with onward influence-driven effects initiated by news media. This finding is consistent with the experimental protocol and illustrates the nature of current debates over “the unvaccinated” (48, 50).

The observation points to the importance of reframing far-reaching discussions into science-based policy rather than feeding the trolls of misinformation (60), albeit no one-size-fits-all vaccination strategy for pandemic preparedness (61). Efficacious debates can help to move science-based policymaking and societal consensus forward, contextualizing the power of social mobilization. As a proven digital contact tracing policy, the national uptake of AliPay HealthCode app in China and TraceTogether app in Singapore had reached nearly 100 and 90%, respectively (42). But early initiatives were met with widespread opposition and rage, as well as masking policy. In the same vein, identifying determinants of vaccine acceptance in various sociocultural contexts and informing vaccination-promoting strategies is a much-needed avenue (46, 62).

## Discussion and Conclusion

In July 2021, the fourth U.S. wave of COVID-19 was frustrating pandemic-weary authorities in the face of people who had refused vaccination. Worse still, Dr. Walensky phrased COVID-19 as a “pandemic of the unvaccinated,” which sparked heated debate among scientists, policymakers, correspondents, and audiences. Some outspoken scientists complained about the political contestation, while others endorsed the official rhetoric. Some policymakers welcome this statement, particularly if they have dealt with a similar domestic status quo. They even inscribed such endorsements in their new orders such as the ORDER-11-18-21 in British Columbia, Canada. Many correspondents had contributed to many of the headlines with allegations, reshaping the populace's values and exacerbating the risk of dualistic antagonism.

The risk of COVID-19 resurgence will persist, making policymaking fraught with challenges (e.g., a top-up dose of vaccine). Enhancing vaccine uptake overcoming vaccine hesitancy is the crux of controversies (63). Arguably, early pronouncements off the mark are inevitably shaping the mindsets of the public and encouraging the vaccinees to play off against the unvaccinated. Branding such rhetoric stigmatizing the unvaccinated could have unintended consequences. Definitely, proven interventions are emerging, including behavioral interventions (64), vaccine passports (51), vaccine incentives and disincentives (56, 65), curated text-messages (42, 66, 67), and vaccine confidence promotion (62).

Authorities must empower scientists and offer a normative framework to underpin science-based policy and practice under the umbrella of moral foundations (68). Our findings offer a cautionary tale about looking beyond open debates to incorporate a consideration of how to inform trust in science around vaccination schemas and non-pharmaceutical interventions (NPIs) (e.g., indoor masks, quarantine, lockdown, digital contact tracing, mHealth interventions). We should take an open mind to appreciate modest introspections and rededications to celebrate medical and societal tenets. Most importantly, scientists, policymakers, correspondents, and audiences should jointly evaluate interventions on the preparedness to identify and fill gaps.

## Data Availability Statement

The datasets for this study can be found in the GitHub repository (https://github.com/Computational-social-science/pandemic_of_the_unvaccinated).

## Author Contributions

ZH conceived the work, reviewed the literature, and wrote the manuscript. ZH and YC analyzed data, reviewed the literature, and edited the manuscript. Both authors contributed to the interpretation of results, manuscript preparation, revisions, read, and approved the final manuscript.

## Funding

This study was partially supported by the National Natural Science Foundation of China (U1936208), the Zhejiang Provincial Natural Science Foundation of China (LZ21F020004), and the Open Research Project of the State Key Laboratory of Media Convergence and Communication, Communication University of China (SKLMCC2021KF015).

## Conflict of Interest

The authors declare that the research was conducted in the absence of any commercial or financial relationships that could be construed as a potential conflict of interest.

## Publisher's Note

All claims expressed in this article are solely those of the authors and do not necessarily represent those of their affiliated organizations, or those of the publisher, the editors and the reviewers. Any product that may be evaluated in this article, or claim that may be made by its manufacturer, is not guaranteed or endorsed by the publisher.
