# Supplementary material for: Open Debates Conducive for Vaccination Rate Flatlines: A Scoping Review and Convergent Cross Mapping
Source: Front Public Health. 2022 Apr 14;10:830933. doi: 10.3389/fpubh.2022.830933 (PMC9047174; doi:10.3389/fpubh.2022.830933)
Supplement: Supplementary file 2 [file Data_Sheet_2.pdf]

Supplementary Material

Final reference list

In the Scoping Review, out of 46 articles identified, 22 articles and 1 preprint in English language were eligible based on exclusion criteria for a full-text review.

1. Bhattacharya A, Ranjan P, Ghosh T, Agarwal H, Seth S, Maher GT, Upadhyay AD, Kumar A, Baitha U, Gupta G, et al. Evaluation of the dose-effect association between the number of doses and duration since the last dose of COVID-19 vaccine, and its efficacy in preventing the disease and reducing disease severity: A single centre, cross-sectional analytical study from India. *Diabetes Metab Syndr Clin Res Rev* (2021) **15**:102238. doi:10.1016/j.dsx.2021.102238
2. Tomori C, Ahmed A, Evans DP, Meier BM, Nair A. Your health is in your hands? US CDC COVID-19 mask guidance reveals the moral foundations of public health. *EClinicalMedicine* (2021) **38**:101071. doi:10.1016/j.eclinm.2021.101071
3. Editorial. US Sanctions on Cuba Further Imperil Global Vaccine Equity. *MEDICC Rev* (2021) **23**: doi:10.37757/MR2021.V23.N3.1
4. Kelly D, Jackson D. Nurses on the wrong side of history: Covid-19 risk minimization, vaccine refusal and social privilege. *J Adv Nurs* (2021) doi:10.1111/jan.15092
5. Garcia M. This Is America: Systemic Racism and Health Inequities Amidst the COVID-19 Pandemic. *Soc Work Public Health* (2021)1–17. doi:10.1080/19371918.2021.1981509
6. Moodley K. COVID-19:'A pandemic of the unvaccinated'? – compassion fatigue among healthcare professionals in South Africa. *South African Med J* (2021) **111**:1040–1041. doi:10.7196/SAMJ.2021.v111i11.16142
7. Kemble S, Edward D, Irvin LH, Pirkle CM. Vaccines and Variants, Valiance and Variance. *Hawai'i J Heal Soc Welf* (2021) **80**:6–9. Available at: http://www.ncbi.nlm.nih.gov/pubmed/34704062
8. Brodhead F. Some Nurses Still Resist Vaccination Against COVID-19. *Am J Nurs* (2021) **121**:15–15. doi:10.1097/01.NAJ.0000798952.99590.bb
9. Badr H, Zhang X, Oluyomi A, Woodard LD, Adepoju OE, Raza SA, Amos CI. Overcoming COVID-19 Vaccine Hesitancy: Insights from an Online Population-Based Survey in the United States. *Vaccines* (2021) **9**:1100. doi:10.3390/vaccines9101100
10. Ramkissoon H. Social Bonding and Public Trust/Distrust in COVID-19 Vaccines. *Sustainability* (2021) **13**:10248. doi:10.3390/su131810248
11. Bennett J. Influenza: Preparing amidst a pandemic. *J Vasc Nurs* (2021) **39**:49. doi:10.1016/j.jvn.2021.07.003
12. Hageman JR. COVID-19: Where Are We Now? *Pediatr Ann* (2021) **50**: doi:10.3928/19382359-20210826-01
13. Wang Y, Ristea A, Amiri M, Dooley D, Gibbons S, Grabowski H, Hargraves JL, Kovacevic N, Roman A, Schutt RK, et al. Vaccination intentions generate racial disparities in the societal persistence of COVID-19. *Sci Rep* (2021) **11**:19906. doi:10.1038/s41598-021-99248-2
14. Hooper VD. Enough Is Enough. *J PeriAnesthesia Nurs* (2021) **36**:443–444. doi:10.1016/j.jopan.2021.09.001

15. Akova M. COVID-19 Vaccination in The Wake of a Fourth Wave of the Pandemic: An Evidence-Based Strategy is Desperately Needed. *Infect Dis Clin Microbiol* (2021) **3**:52–54. doi:10.36519/idcm.2021.82
16. Cohen AB. In the September 2021 Issue of the Quarterly. *Milbank Q* (2021) **99**:605–609. doi:10.1111/1468-0009.12541
17. Yu F, Lau L-T, Fok M, Lau JY-N, Zhang K. COVID-19 Delta variants—Current status and implications as of August 2021. *Precis Clin Med* (2021) **15**:102238. doi:10.1093/pcmedi/pbab024
18. Longhurst JM, Thier M. Relatively Good News Amid Covid-19. *Rural Educ* (2021) **42**:99–103. doi:10.35608/ruraled.v42i2.1220
19. Franco JVA. Should COVID-19 vaccination be mandatory? *BMJ Evidence-Based Med* (2021)bmjebm-2021-111813. doi:10.1136/bmjebm-2021-111813
20. Samaranayake L, Fakhruddin KS. Pandemics past, present, and future: Their impact on oral health care. *J Am Dent Assoc* (2021) **152**:972–980. doi:10.1016/j.adaj.2021.09.008
21. Hamm R, Weiner R, Corcoran MP. How to Transcend the Researcher/Researched Divide in the Social Sciences: Reflections on the Contribution of Collective Memory-Work. *SSRN Electron J* (2021) doi:10.2139/ssrn.3935390
22. Kampf G. COVID-19: stigmatising the unvaccinated is not justified. *Lancet* (2021) **398**:1871. doi:10.1016/S0140-6736(21)02243-1
23. Olivier B. Therapeutic reflections on the ‘pandemic.’ *Preprints* (2021) Available at: https://www.researchgate.net/publication/355980600_Therapeutic_reflections_on_the_’pandemic’

Table S1 Summary of reviewed studies.

#	Author and publication year	Document Type	Study objectives	Methods	Study location	Viewpoints identified
1	Bhattacharya et al. (2021)	Original article	To assess the dose-effect association between COVID-19 vaccination and probability of turning RT-PCR positive and to assess the correlation between disease severity and vaccination status.	cross-sectional study	India	The authors backed up the claim that one of the world’s most developed countries, the United States, is dealing with a ‘pandemic of the unvaccinated,’ citing the local media *Times of India*.
2	Tomori et al. (2021)	Commentary	To call for a renewed commitment to core public health principles of collective responsibility, health equity, and human rights.	Critical review	USA	The authors criticized that the CDC Director for referring to a “pandemic of the unvaccinated,” implying that those who become ill are to blame for their own suffering and that their deaths are tolerable because they could have been vaccinated. These moral deficiencies are symptomatic of a broader for collective responsibility, equity, and human rights in US

						public health policy. And the moral foundations of public health must be considered in CDC guidance, which should provide a normative framework to support public health policy and practice.
3	The Editors (2021)	Editorials	To editorialize about how US sanctions against Cuba further endanger global vaccine equity.	Editorials	NA	The Editors editorialized on the more virulent Delta variant, which posed a risk to the 'pandemic of the unvaccinated' before herd immunity was achieved, citing NPR news entitled "U.S. COVID Deaths Are Rising Again. Experts Call It A 'Pandemic of the unvaccinated'."
4	Kelly and Jackson (2021)	Editorials	To editorialize on COVID-19 risk minimization, vaccine refusal and social privilege among nurses.	Editorials	UK, Australia	The authors criticized the fact that COVID-19 is increasingly being referred to as a disease of the unvaccinated, since that many people have not even had the opportunity to be vaccinated, and some who are now seriously ill with COVID-19 chose to remain unvaccinated. They were adamant that healthcare workers (HCWs) get their jabs.
5	Garcia M. (2021)	Article	To identify and describe the systemic racism and health inequities amidst the COVID-19 pandemic in America.	Critical review, Syndemics theory	USA	The author criticized the U.S. CDC for warning that the highly contagious COVID-19 Delta variant had spread due to a "pandemic of the unvaccinated," emphasizing that people had not followed the CDC's recommendations for getting vaccinated or engaging in community mitigation activities. He criticized the CDC for failing to understand the biological-social implications of systemic racism before reaching such a conclusion.
6	Moodley K. (2021)	Editorials	To editorialize on compassion fatigue among healthcare professionals in South Africa.	Editorials	South Africa	The author argued that the false descriptions of COVID-19 as a 'pandemic of the unvaccinated' or 'a self-inflicted pandemic' are simply attributing the current outbreak to the unvaccinated, regardless of medical contraindications, and such stigma would further encourage selective treatment behaviors in low-and-middle-income countries (LMICs),

						against the medical doctrine of sacrificial expectations.
7	Kemble et al. (2021)	Commentary	To comprehend the issues surrounding vaccines and variants, valiance and variance.	Critical review	USA	The authors backed up the claim that the United States is grappling with a 'pandemic of the unvaccinated,' citing *The Times of India* news entitled with "C.D.C. director warns of a 'pandemic of the unvaccinated'."
8	Brodhead F. (2021)	News Item	To report the news on some nurses still resist vaccination against COVID-19.	News report	USA	The author neutrally reported that top concerns of unvaccinated nursery staff were safety, including uncertainty about the vaccines' long-term effects, and mistrust of the vaccine development and approval process, according to a survey by the American Nurses Association (ANA).
9	Badr et al. (2021)	Article	To identify individual-level determinants of COVID-19 vaccine hesitancy based on the Health Belief Model (HBM) and Theory of Planned Behavior (TPB).	Cohort study	USA	The authors endorsed the official rhetoric – "COVID-19 has evolved into a pandemic of the unvaccinated."
10	Ramkissoon H. (2021)	Communication	To discuss the role of social bonds and public trust/distrust and word of mouth communication in vaccine decision making.	Critical review, Commentary	UK, Norway, South Africa	The author endorsed the official rhetoric – "The COVID-19 pandemic in the United States is now being called the pandemic of the unvaccinated, mainly due to the Delta variant."
11	Bennett J. (2021)	Editorials	To editorialize on influenza amidst COVID-19 pandemic.	Editorials	USA	The Editor-in-Chief of *Journal of Vascular Nursing* endorsed the official rhetoric – "COVID–19 surges are more pronounced in low vaccination areas becoming a pandemic of the unvaccinated."
12	Hageman JR. (2021)	Editorials	To editorialize the issues surrounding vaccine-associated myocarditis and pericarditis, Delta variant, and in-person school.	Editorials	USA	The Editor-in-Chief of *Pediatric Annals* endorsed the official rhetoric of Dr. Walensky, the U.S. CDC Director.
13	Wang et al. (2021)	Article	To ascertain the effects of racial disparities in the	Simulation	USA	The authors concluded that communities with more Black and Latinx residents had fewer

			inclination to become vaccinated on continued infection rates and the attainment of herd immunity.			people who were initially willing to be vaccinated. And they critiqued that Tate Reeves, the governor of Mississippi, who acknowledged that the 4th wave of pandemic is becoming a ‘pandemic of the unvaccinated’, spurred by the surge of Delta variant in Mississippi.
14	Hooper VD. (2021)	Editorial Opinion	To editorialize vaccination among nurses.	Editorials	USA	The author strongly urged that healthcare workers (HCWs) should get their jabs, especially nurses.
15	Akova M. (2021)	Editorials	To editorialize how to increase public confidence about vaccination schedules offered and decrease vaccine hesitancy in the communities.	Editorials	Turkey	The Editor-in-Chief of *Infectious Diseases and Clinical Microbiology* applauded the statement “the pandemic of the unvaccinated” for its appropriateness, even though fully vaccinated populations could also become infected with high viral loads and there is not a one-size-fits-all evidence-based vaccination strategy.
16	Cohen AB. (2021)	Editorials	To editorialize the September 2021 issue of *The Milbank Quarterly*.	Editorials	USA	The editor of *The Milbank Quarterly* commented that vaccine hesitancy in the US nudged COVID-19 into a pandemic of the unvaccinated, despite the availability of effective vaccines.
17	Yu et al. (2021)	Short communication	To discuss the virologic aspect, clinical implications, and public health implications, and provide recommendations to health authorities.	Critical review, Commentary	USA, China	The authors strongly suggested that governments should persuade all citizens to take their shots since the present pandemic has evolved into an unvaccinated pandemic.
18	Longhurst and Thier (2021)	Policy brief	To document how schools were serving various vulnerable subgroups among student populations.	Commentary	USA	The authors concluded that interregional differences of vaccine uptake in USA were the primary cause of an unvaccinated pandemic.
19	Franco JVA. (2021)	Editorials	To editorialize should COVID-19 vaccination be mandatory.	Editorials	Argentina	The Editor-in-Chief of *BMJ Evidence-Based Medicine*, advocated for vaccine mandates to lift vaccination rate flatlines in high-income countries (HICs) with sufficient supply, as an essential component of preparedness against vaccine hesitancy and anti-vaxxer propaganda.

20	Samaranayake and Fakhruddin (2021)	Invited review	To review the history of pandemics, the probable reasons for their emergence, and the COVID-19 pandemic due to SARS-CoV-2 and its variants, as well as its possible impact on dentistry during the post-pandemic period.	Review	Hong Kong, United Arab Emirates	The authors concluded that the high efficacy of the approved COVID-19 vaccines, extrapolated from the dubbed 'pandemic of the unvaccinated' made by some Western officials.
21	Hamm et al. (2021)	Working paper	To present a general briefing on Collective Memory-Work.	Review, Commentary	Ireland	The authors slammed such political contestation that excoriated the unvaccinated in absence of grounded evidence.
22	Kampf G. (2021)	Correspondence	To response to the article entitled "How the unvaccinated threaten the vaccinated for COVID-19: a Darwinian perspective" in the *Proc Natl Acad Sci USA*.	Critical review, Commentary	Germany	As one of the first voices, the author urged that authorities of the USA and Germany put extra effort into society together rather than stigmatizing the unvaccinated.
23	Olivier B. (2021)	Preprint	To gain insight into the contradictory aspects of the present so called 'pandemic', with a view to arriving at a cogent notion of what 'psychotherapy' would mean under these circumstances.	Critical review, Commentary	South Africa	The author denounced that some authorities pitted the vaccinees against the unvaccinated with specious statements, regardless of the fact that the virus mutates in the vaccinees.
